# Antitumor Immunity Is Controlled by Tetraspanin Proteins

**DOI:** 10.3389/fimmu.2018.01185

**Published:** 2018-05-29

**Authors:** Fleur Schaper, Annemiek B. van Spriel

**Affiliations:** Department of Tumor Immunology, Radboud Institute for Molecular Life Sciences, Radboud University Medical Center, Nijmegen, Netherlands

**Keywords:** tetraspanins, antitumor immunity, tumor microenvironment, adaptive immunity, innate immunity

## Abstract

Antitumor immunity is shaped by the different types of immune cells that are present in the tumor microenvironment (TME). In particular, environmental signals (for instance, soluble factors or cell–cell contact) transmitted through the plasma membrane determine whether immune cells are activated or inhibited. Tetraspanin proteins are emerging as central building blocks of the plasma membrane by their capacity to cluster immune receptors, enzymes, and signaling molecules into the tetraspanin web. Whereas some tetraspanins (CD81, CD151, CD9) are widely and broadly expressed, others (CD53, CD37, Tssc6) have an expression pattern restricted to hematopoietic cells. Studies using genetic mouse models have identified important immunological functions of these tetraspanins on different leukocyte subsets, and as such, may be involved in the immune response against tumors. While multiple studies have been performed with regards to deciphering the function of tetraspanins on cancer cells, the effect of tetraspanins on immune cells in the antitumor response remains understudied. In this review, we will focus on tetraspanins expressed by immune cells and discuss their potential role in antitumor immunity. New insights in tetraspanin function in the TME and possible prognostic and therapeutic roles of tetraspanins will be discussed.

## Introduction

It is now well known that the immune system plays an important role in preventing tumor formation, growth, and metastasis. This is exemplified by the increased susceptibility of immunocompromised patients to develop cancer, and by the recent success of novel cancer immunotherapies including checkpoint inhibitors, dendritic cell (DC) vaccination, and chimeric antigen receptor T cells, which demonstrate that the immune system can be harnessed against cancer.

**Figure 1:**
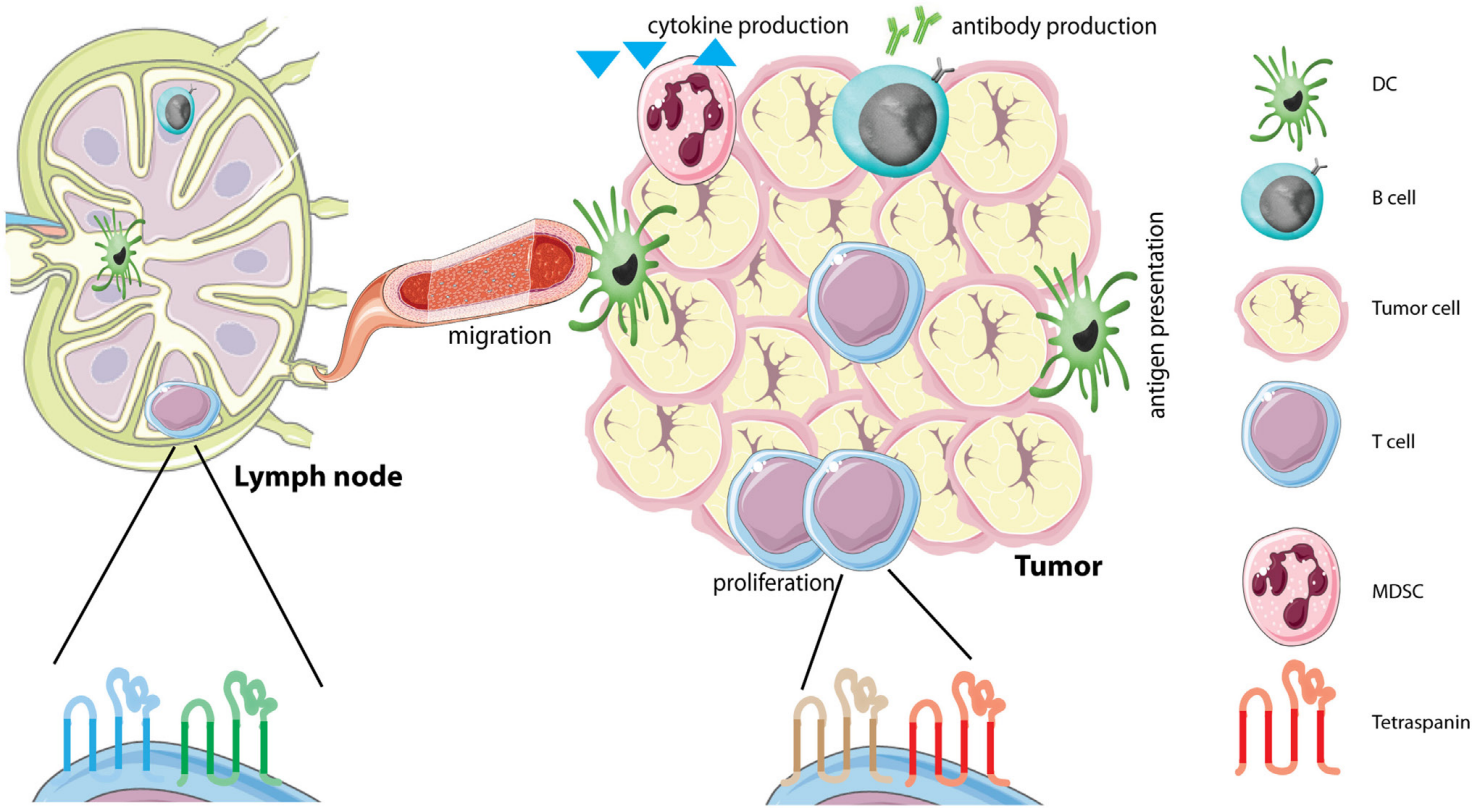
Tetraspanins on immune cells in the tumor microenvironment (TME). The TME is comprised of several different cells, including tumor cells and different immune infiltrating cells. Immune cells in the TME produce different soluble factors, including cytokines or antibodies. Each immune cell subset contains its own distinct tetraspanin web composed of tetraspanins that can have either stimulating or inhibitory functions (see Table 1). We hypothesize that immune cells alter the composition of the tetraspanin web when they migrate from the lymph node to the tumor caused by the immunesuppressive environment of tumors. Abbreviations: DC, dendritic cell; MDSC, myeloid-derived suppressor cell.

Antitumor immunity is dependent on tumor cell uptake by antigen-presenting cells (APCs) (DCs, macrophages) that subsequently migrate to nearby lymph nodes to activate T and B cells. After clonal expansion, antigen-specific CD8 T cells can migrate toward the tumor, aiming to destroy tumor cells, a process called tumor immunosurveillance. However, this process is far from perfect as non-immunogenic variants of the tumor escape, resulting in tumor recurrence (1, 2). Escape from the immune system can also occur in other ways, for instance, by inducing an immunosuppressive state in the tumor microenvironment (TME) (3, 4). Here, tumors create a niche where they recruit different cell types to create a specific microenvironment, which favors tumor growth and metastasis (5). These cells include immune cells, which often have acquired immunosuppressive properties, such as regulatory T cells (Tregs), tumor-associated macrophages, and myeloid-derived suppressor cells (MDSCs) (6, 7) (Figure 1). Tumor-associated macrophages are very plastic cells that can adapt their phenotype in response to different tumor cell products or hypoxia (8). Alterations in cellular phenotypes are often accompanied by membrane protein reorganization, as different membrane receptors will be upregulated or internalized.

Tetraspanins, or transmembrane-four superfamily proteins, are evolutionary conserved membrane organizers that regulate protein trafficking, adhesion, migration, fusion, and signaling (9–12). While many tetraspanins (CD9, CD81, CD151) are widely expressed, others are restricted to hematopoietic cells (CD37, CD53, Tssc6). Tetraspanins are not constitutively expressed on all cell types and can differ between effector fates (13–15). Tetraspanins can interact with each other and with partner proteins on the same cell whereby they form “tetraspanin-enriched microdomains” (TEMs) or “tetraspanin web” (9, 11, 16). The overall view is that tetraspanins are modulators of signal transduction, providing organization to membrane domains through lateral interaction with their partners (11, 17–23), including integrins [reviewed in Ref. (24)] and immunoreceptors [CD19 (25), MHC class molecules (17, 18)]. The immunological importance of these interactions has been demonstrated in multiple tetraspanin-deficient (−/−) mice (CD37, CD53, CD81, CD82, Tssc6, CD151) that have defects in humoral and/or cellular immune responses (14, 26–29). These defects include cell migration, T cell proliferation (27, 30–32), antibody production (25, 33), and antigen presentation (14, 34–36). As such, it is likely that they also control antitumor immunity. While studies have clearly demonstrated effects of tetraspanins in primary tumor progression [reviewed in Ref. (37)] or metastasis [reviewed in Ref. (38)], detailed analyses of antitumor immunity in tetraspanin −/− mice is still scarce.

Studies on human tumor cells reported associations between tetraspanin expression and tumor progression showing both reduced (CD82, CD9) and increased expression (CD151, Tspan8) in various cancer types (12, 15, 37–47). In patients with invasive breast cancer, it was shown that CD9 on immune cells was associated with a longer disease free survival, while CD9 expression on the tumor cells showed the opposite effect (48). The relevance of CD37 in tumor suppression has been recently shown in CD37^−/−^ mice that spontaneously develop B-cell lymphoma, and in patients with CD37-negative B-cell lymphoma that have poor survival (45, 49). These results are in line with studies that report tetraspanin expression to serve as a prognostic marker for cancer patients (50). In addition, these findings indicate that tetraspanins not only influence immune cell signaling but also directly protect from tumor formation. This review focuses on tetraspanins expressed on immune cells, and their possible role in antitumor responses and the TME.

## Tetraspanins and Antigen Presentation

The first steps in an antitumor immune response are the uptake, processing, and presentation of tumor antigens by APCs. DCs are the most professional APCs, and infiltration of mature activated DCs into tumors has been associated with increased patient survival. There is also ample evidence that tumors can inhibit APCs, leading to escape from antitumor responses (51). CD53, CD81, CD82, and CD37 have been shown to associate with MHC class II complexes (17, 52–54). Recently, CD9 was reported to be involved in MHC class II trafficking in human monocyte-derived DCs (35). The functional consequences of these interactions are demonstrated by altered antigen presentation capacity of tetraspanin^−/−^ DCs. Both CD37^−/−^ and CD151^−/−^ DCs were hyperstimulatory to CD4 and CD8 T cells, although by different underlying mechanisms. CD151 was involved in inhibiting co-stimulation, while absence of CD37 led to increased peptide presentation (34). In Tccs6xCD37 double knock-out mice, an exaggerated hyperstimulatory phenotype of DCs was observed compared to DCs of single knock-out mice (55). This study indicates complementary functions for these two tetraspanins. In addition, DCs lacking CD82 had defects in processing MHC class II (14). Tetraspanin function in cross-presentation (the presentation of extracellular antigens in the context of MHC class I) by DCs has not been investigated, but is not unlikely considering that CD53, CD81, and CD82 interact with MHC class I molecules [(17), and own unpublished data]. Moreover, DCs lacking CD82 showed defective DC–T conjugate formation (14), and CD81 was found enriched in the contact area between APCs and T cells (56), supporting a function for tetraspanins in immunological synapse formation. Finally, tetraspanins (CD63, CD9, CD81, CD37) on exosomes may influence antigen presentation possibly *via* transfer of MHC–peptide complexes (57, 58). CD63 has been reported to inhibit antigen presentation as CD63 knockdown in APCs demonstrated increased secretion of exosomes containing MHCII (59). Together, these studies show that tetraspanins control antigen presentation either at the level of MHC–T cell receptor (TCR) interactions, at the level of co-stimulation, or *via* exosomes, which likely has implications for antitumor responses.

## Tetraspanins and Immune Cell Motility

To mount an adequate immune response, immune cells need to migrate from peripheral tissues to draining lymph nodes and to the site of the tumor. It is well known that tetraspanins interact with multiple different integrins and as such influence the migratory capacity of cells (60). In the immune system, absence of CD151 was found to decrease T cell motility, leading to reduced inflammation in a model for inflammatory bowel disease (61). Trafficking of DCs to lymph nodes has been studied in different tetraspanin-deficient mice. CD37^−/−^ mice challenged with two different doses of an immunogenic tumor showed defective tumor rejection compared to wild-type (WT) mice, indicating that CD37 is directly involved in antitumor immunity (62). Using irradiated tumor cells, it was shown that T cell responses were impaired, which was due to impaired DC migration to the draining lymph nodes (62). A different study confirmed the decreased motility of CD37^−/−^ DCs (14) and neutrophils (63), and increased motility of CD82^−/−^ DCs (14). Interestingly, the functional effects of CD82 are opposite to those of CD37 indicating that these tetraspanins counteract each other (14). Furthermore, CD81 was reported to be important in DC migration and formation of membrane protrusions *in vitro* (64). The underlying molecular mechanism involved cytoskeleton rearrangements *via* regulation of Rac-1 and RhoA, small GTPases that regulate the actin network. CD81 was required for Rac-1 activation (65), CD82 negatively regulated RhoA, and CD37 promoted activation of Rac-1 (27). Moreover, CD37, CD81, and CD82 have all been reported to interact with integrins (24, 33, 52, 63, 66), and although leukocytes are not dependent on integrins for migration in 3D environments (67), this may provide an additional mechanism for tetraspanin involvement in 2D migration. These studies show that tetraspanins are important in immune cell migration, thus making it likely they are also involved in leukocyte migration into the TME.

## T and B Cell Activationl and Proliferation

Activation of T cells depends on antigen recognition presented in MHC–peptide complexes on the surface of APCs during immunological synapse formation. Recently, it was determined that CD9 and CD151 support integrin-mediated signaling at the immunological synapse in T cells (68). Accordingly, CD81 in T cells was involved in the organization of the immunological synapse by interacting with ICAM-1 and CD3 (69).

Antigen-presenting cell–T cell interaction and subsequent engagement of TCR and co-stimulatory molecules leads to naive T cell activation and proliferation (70), which can occur in the nearby lymph nodes or the TME. It is well-known that tumor-infiltrating lymphocytes can be an important prognostic factor for cancer (71). More specifically, CD8 cytotoxic T cells are associated with favorable patient outcome while Tregs are associated with decreased survival (6, 72). Furthermore, the positive effect of immune checkpoint inhibitors on clinical outcome of patients with melanoma or lung carcinoma shows that exhausted/dysfunctional T cells in the TME may be reactivated by anti-PD-1 therapy (73, 74). Taken together, these studies underline the importance of T cells in anti-tumor immunity.

Different tetraspanins have been linked to T cell proliferation, as CD37^−/−^, CD151^−/−^, Tssc6^−/−^, and CD8^−/−^ T cells were all hyperproliferative upon TCR stimulation (27, 30–32). Moreover, double CD37^−/−^ Tssc6^−/−^ mice displayed an exaggerated hyperproliferative T cell response, and impaired formation of antigen-specific CD8^+^ T cells after infection (55). In contrast, CD151-positive human T cells exhibited increased proliferation compared to CD151-negative T cells (13).

Another important facet of T cell biology is differentiation into different sub-types. CD81^−/−^ mice displayed impaired Th2 responses, possibly linking tetraspanins to T cell differentiation (32, 75–77). These mice fail to develop Th2-dependent allergic airway hyperreactivity (77). *In vitro* studies revealed that altered B–T cell interactions were responsible for the deficient Th2 response (76). These studies should be further expanded to investigate different T cell subsets, Th1-Th2 balance, and especially T cells in the TME (such as Tregs or Th17 cells) to unravel tetraspanin function in T cell differentiation during antitumor immunity.

A recent study with CD81^−/−^ mice elegantly demonstrated that tumor growth and metastasis were severely impaired in CD81^−/−^ mice compared to WT mice. Both Tregs and MDSCs lacking CD81 were observed to be deficient in their suppressive ability (46). This is one of the first studies investigating the effect of tetraspanins on regulatory immune cells.

B cells have a dual role in tumor immunity as they can both inhibit and stimulate tumor growth. Tumor-specific antibodies produced by B cells can opsonize tumor cells and lead to antibody-dependent cytotoxicity by natural killer (NK) cells and phagocytes. On the other hand, regulatory B cells (Bregs) secrete IL-10 and TGFβ, which directly inhibit effector immune cells, thus suppressing antitumor immunity (78). Tetraspanins function in humoral immunity has been evidenced by different studies in tetraspanin-deficient mice and the first documentation of a CD81-deficient patient. CD81 is important in the trafficking of CD19, part of the co-receptor complex of the B cell receptor (BCR), to the surface of B cells (25). BCR signaling occurs when B cells encounter their antigen and is important for B cell proliferation, survival, and antibody production. Absence of CD81 led to CD19-deficiency resulting in antibody defects in mice (32, 76) and humans (25). CD81^−/−^ mice show impaired B cell proliferation, decreased responses to Th2 stimuli, decreased antibody production (32), and impaired B cell proliferation after BCR activation (26). CD53, although not required for CD19 expression, is also important for B cell function, as we recently discovered that CD53 promoted BCR-dependent protein kinase C (PKC) signaling (19). Both human and murine CD53^−/−^ B cells have defects in translocation of PKC to the plasma membrane, consistent with an elegant study demonstrating that CD82 stabilizes PKC activation at the surface of leukemia cells (60). These findings indicate that tetraspanins can directly influence immune cell signaling. CD37 is highly expressed by B cells (79) and controls antibody production as shown in CD37^−/−^ mice that display decreased IgG and increased IgA levels, which is a B-cell intrinsic phenotype (29, 80). The underlying mechanism involved CD37 regulation of α4β1 integrin-Akt signaling, which is required during follicular DC–B cell interactions and supports survival of IgG1-secreting cells (33). The importance of CD37 in B cell survival has been confirmed in an independent study demonstrating that the cytoplasmic domains of CD37 couple to the Pi3K–Akt survival pathway (81). Tetraspanin function in anti-tumor immunity is further supported by our unpublished findings showing increased tumor growth in CD53^−/−^ mice compared to WT mice using a syngeneic immunogenic tumor model (F. Schaper et al., in preparation), which is in accordance with the impaired anti-tumor immunity observed in CD37^−/−^ mice (62).

To conclude, tetraspanins have crucial functions in both T and B cell proliferation, survival, and signaling. It is interesting that these functions are non-redundant (deficiency of one tetraspanin results in a certain phenotype) and specific (for example CD81 controls CD19, whereas CD37 controls α4β1 integrin on B cells). Given these specific functions of tetraspanins and their partner molecules, it stands to reason that this can result in either an antitumor or pro-tumor response mediated by immune cells in the TME.

## Cytokine Production and Other Effector Functions

Cytokines are a central part of cellular communication and stimulate cell migration to sides of inflammation. These cytokines can be either immunosuppressive (IL-10, TGFβ, or IL-35) or proinflammatory (IL-12, γIFN, TNFα). Several studies demonstrated that tetraspanins can influence cytokine production. CD37 has been reported to inhibit IL-6 signaling upon infection (80, 82, 83), and during lymphomageneses (45). CD53 has been implicated as negative regulator of IL-6, TNFα, and IL-1β in a population study of house dust mite (84) and linked to TNF-α by genome-wide association studies (85). In line with this, CD81^−/−^ DCs produced more TNF-α compared to WT DCs upon *Listeria* infection (86). Additionally, TNF-α production was increased by CD9^−/−^ macrophages compared to WT macrophages after stimulation with LPS (87). CD9 has also been linked to production of TGFβ and IL-10. Bregs are known to produce large amounts of IL-10 and two independent mouse studies discovered that CD9 could serve as novel phenotypic marker for Bregs (88, 89). Although CD9^−/−^ mice do not have aberrant B cell development or humoral immunity (90), Breg presence and function in CD9^−/−^ mice has not been investigated to date.

In a small study of patients with metastatic melanoma, CD9 expression on NK cells was observed to correlate strongly with serum levels of TGFβ (91). Interestingly, CD9 was absent on NK cells in healthy controls, but upregulated after incubation with TGFβ (91, 92). These CD9-positive NK cells are normally found in the maternal part of the placenta (93), where they exert immunosuppressive actions. These data indicate that suppressive factors, which are also found in the TME, can alter tetraspanin expression on immune cells, which has immunological consequences.

## Future Directives: Tetraspanins on Immune Cells in the TME

Tetraspanins are emerging as important organizing proteins on immune cells that control both humoral and cellular immune responses (Table 1), by either stimulating or inhibiting immune cell function. However, more insight is needed into understanding tetraspanins on immune cells in the TME including regulatory lymphocytes, MDSCs, and tumor-associated macrophages. Recently, CD81 has been demonstrated to control MDSC and Treg function in a murine tumor model, which is the first *in vivo* evidence of tetraspanin function in antitumor immunity (46). This, together with the finding that CD37^−/−^ mice have impaired antitumor responses (62) indicates that tetraspanins directly contribute to antitumor immunity. However, tetraspanin expression has thus far only been studied on immune cells from blood or bone marrow (79), and not yet in the TME. In this TME, both stimulating and suppressive immune cells are present, each with their own distinct tetraspanin web. We propose a model in which tumor cells have the ability to alter the composition of the tetraspanin web on immune cells that enter the TME from the circulation (Figure 1). Tumor environmental factors that may influence tetraspanin expression on immune cells include suppressive cytokines, low oxygen levels (hypoxia), growth factors, damage-associated molecular patterns (DAMPs) and tumor-immune cell contact. These tumor environmental factors may either increase or decrease the expression of different tetraspanins on immune cells. Plasticity of immune cells plays an important role in the TME, and it is well established that immune cells can change their phenotype by altering membrane protein expression. Since altered (increased or decreased) tetraspanin expression affects immune cell function, we hypothesize that a changed tetraspanin web supports immune cell plasticity through altered membrane protein organization. This model is supported by (1) multiple studies demonstrating that tetraspanin-deficiency affects proliferation, migration, cytokine production, and antigen presentation, (2) CD9 upregulation on NK cells after TGFβ incubation leading to immunosuppressive NK cells (91), (3) regulation of TGFβ by tetraspanins (94, 95), and (4) own unpublished observations demonstrating that human lymphocytes cultured with tumor cells change expression of multiple tetraspanins. We are only at the beginning of understanding the dynamic nature of the tetraspanin web on immune cells, and we envisage that its composition will change depending on the tumor state (elimination, equilibrium, or escape). Using multispectral imaging (79, 96) on resected tumor material from patients, effects on tetraspanin expression can be investigated in the future. We anticipate that targeting tetraspanins on specific immune subsets (such as CD9 on Bregs) in the TME may have therapeutic potential. Hereby, it should be taken into account that the same tetraspanin can stimulate or inhibit immune cell function, depending on the immune cell type it is expressed on. Research investigating tetraspanins as therapeutic targets in cancer is already ongoing (50), exemplified by targeting CD37 in clinical trials for B cell malignancies (97, 98).

**Table 1:** Key functions of individual tetraspanins on immune cell subsets.

Tetraspanin	Function on immune cells
CD37	− T cell proliferation, peptide MHC presentation, antibody production, IL-6 signaling by B cells
	+ Dendritic cell (DC) migration, B cell survival

CD53	+ B cell receptor-dependent protein kinase C signaling
	− IL-6, TNFα production

Tssc6	− T cell proliferation

CD9	− TNF-α production
	+ MHC class II trafficking

CD81	− T cell proliferation, TNF-α production
	+ DC motility, immunological synapse organization, Th2 response, antibody production

CD82	− DC migration
	+ DC–T cell conjugation
	+ Antigen presentation

CD151	− T cell proliferation, T cell motility, co-stimulation in DCs

CD63	− exosome secretion

*−, inhibits; +, stimulates*.

Taken together, further investigation into tetraspanin function on immune cells will add to our understanding of the role that these membrane proteins play in antitumor immunity and the possibility to target the tetraspanin web on immune cells in the TME.

## Author Contributions

AS supervised the study and FS and AS both wrote the manuscript.

## Conflict of Interest Statement

The authors declare that the research was conducted in the absence of any commercial or financial relationships that could be construed as a potential conflict of interest.
